# Investing in Addressing NCDs and Mental Health Conditions: a Political Choice

**DOI:** 10.5334/aogh.4649

**Published:** 2025-04-29

**Authors:** Téa E. Collins, Amanda Karapici, Daria Berlina

**Affiliations:** 1 World Health Organization, Avenue Appia 20, 1211 Geneva, Switzerland

**Keywords:** Noncommunicable disease, UN high‑level meetings, political will, multisectoral action, financing, integrated care, investments

## Abstract

Noncommunicable diseases (NCDs) and mental health conditions are responsible for 75% of deaths globally, with the greatest burden in low‑ and middle‑income countries (LMICs). The economic impact of NCDs and mental health conditions on households, health systems, and economies is also staggering. Despite the growing burden of NCDs, the available funding to address these diseases is limited, with less than 2.3% of global health development assistance spent on NCDs. The 2025 United Nations (UN) High‑Level Meeting on NCDs will provide a critical opportunity to reaffirm global commitments, enhance political will, and advocate for greater resource mobilization for the prevention and control of NCDs and mental health conditions. Investments will be needed in the strengthening of health systems, integrated models of care, multisectoral action, and a greater focus on vulnerable populations. Increased domestic and international funding will be required for implementation research as well, to ensure sustainable progress toward overcoming context‑specific barriers impeding the achievement of Sustainable Development Goals (SDG) target 3.4 on reducing premature mortality from NCDs and improving mental health and well‑being. The challenge remains to convert high‑level commitments into actionable, measurable strategies and mobilize the resources required to meet these goals, particularly in low‑income settings.

## Introduction

Noncommunicable diseases (NCDs) are a major global health and sustainable development challenge resulting in 43 million or 75% of all global non‑pandemic‑related deaths in 2021 [1]. Every 2 seconds, someone dies from NCDs without reaching 70 years of age [1]. Each year, 14.76 million deaths out of 18 million premature deaths take place in low‑ and middle‑income countries (LMICs) [2]. Even though the likelihood of dying prematurely from any of the four primary NCDs (cardiovascular diseases, cancer, diabetes, and chronic respiratory diseases) decreased from 2000 to 2019, progress has been slow [1].

The economic impact of NCDs and mental health conditions on households, health systems, and economies is also staggering. In 2019, the direct medical costs of NCDs in the Gulf Cooperation Council (GCC) countries were estimated at USD 16.7 billion, equal to approximately 0.6% of the gross domestic product (GDP) of these countries, with productivity losses adding another USD 80 billion [3]. Similarly, in the Caribbean, NCDs have an economic impact estimated between 1.36% and 8% of GDP, excluding indirect costs such as lost productivity [4]. In South America, the cumulative economic loss due to NCDs and mental health conditions is projected to reach approximately USD 7.3 trillion between 2020 and 2050, accounting for about 4% of the region’s total GDP [5].

Between 27 million and 50 million of the world’s poorest people spend more than 40% of their income (defined as a catastrophic health expenditure to pay out‑of‑pocket each year) in direct costs for health services for NCDs [6]. Accumulating evidence indicates that the smaller but more frequent out‑of‑pocket expenditures on outpatient healthcare, especially medicines, drive out‑of‑pocket spending. The total number of people experiencing out‑of‑pocket health spending has continuously increased globally since 2000 and surpassed 1 billion by 2019 [7].

Despite their destructive long‑term impact on people and economies, NCDs and mental health conditions continue to be the most underfunded global health areas. There is a significant mismatch between the healthcare needs of people living with NCDs and mental health conditions, especially in LMICs, and the resources allocated to address them. The share of the development assistance for health dedicated to NCDs is also low. In 2023, it remained at less than 2.3% or USD 64.6 billion of total development assistance for health [8].

According to the UN General Assembly report on “*Progress on the prevention and control of non‑communicable diseases and the promotion of mental health and well‑being*” [1], the economic burden of NCDs and mental health conditions is worsened by inadequate investment in prevention and treatment, as many countries face financial constraints, leading to underinvestment in these services and limited ability to implement policies addressing their risk factors.

Countries vary in their ability to address the growing burden of NCDs and mental health conditions. The challenging areas requiring urgent attention include the lack of political will; the lack of multisectoral policies, plans, and coordination mechanisms for whole‑of‑government and whole‑of‑society actions; difficulty in setting priorities; weak health systems; the impact of social, economic, and commercial determinants; insufficient technical and operational capacities to accelerate action across sectors and stakeholders; and a lack of domestic and international investments to scale up interventions for NCD prevention and control [9].

Investing in preventing and controlling NCDs and mental health conditions is vital to reduce the burden of disease, and fight poverty and inequalities [10]. Sustainable funding to address NCDs and their risk factors is also important to strengthen health systems and achieve Universal Health Coverage (UHC) and broader Sustainable Development Goals (SDGs) and targets. Without such investments, countries will continue falling behind, and SDG target 3.4 for the one‑third reduction in premature mortality from NCDs and mental health will not be achieved.

This viewpoint article highlights the urgent need for increased investment in the prevention and control of NCDs and mental health conditions, positioning this as a critical political and economic decision rather than solely a health concern. We argue that reversing the chronic underfunding of interventions to prevent and control NCDs and mental health conditions requires strong political commitment, improved multisectoral action, and strategic health system investments in priority areas with a focus on vulnerable populations. We emphasize that, while numerous global commitments have been made, mostly through the United Nations General Assembly High‑Level Meetings (UNGA HLMs) for NCDs, there remains a persistent gap in translating these commitments into actionable, well‑funded national policies and programs. Addressing this gap is essential to achieving SDG 3.4 and reducing the global NCD burden.

## The UNGA High‑Level Meetings, Events, and Commitments for NCD Prevention and Control: Generating Global Political Momentum

The fourth UNGA HLM for NCDs, scheduled for September 2025, represents a critical opportunity for world leaders, health advocates, and other stakeholders to evaluate progress and reaffirm their commitments to combating NCDs to achieve SDG target 3.4. Previous high‑level meetings have played a significant role in laying the groundwork for international action and cooperation.

The first UNGA HLM on NCD prevention and control, held in 2011, marked a milestone by bringing attention to these diseases globally. It resulted in the adoption of a political declaration recognizing NCDs as a significant challenge for development in the 21st century and triggered the formulation of the World Health Organization (WHO) Global Action Plan for the Prevention and Control of NCDs, as well as the setting of global voluntary targets to address these diseases and their risk factors.

The 2011 HLM, and the subsequent HLMs in 2014 and 2018, reinforced the need for increased policy coherence to scale up multisectoral and multistakeholder action and additional investments to address NCDs, their shared risk factors, and underlying social, economic, and environmental determinants, leading to a myriad of mainly WHO‑led high‑profile initiatives (Table 1).

**Table 1:** Major United Nations high‑level events/commitments to investing in NCD prevention and control and enhancement of mental health and well‑being.

YEAR	EVENT	EXAMPLES OF FINANCING‑RELATED HIGH‑LEVEL COMMITMENTS AND ACTIONS
**2011**	First UNGA High‑Level Meeting on NCD Prevention and Control	In the Political Declaration, governments committed to “identify[ing] and mobiliz[ing] adequate, predictable and sustained financial resources…for long‑term financing of NCD prevention and control.”
**2013**	WHO Global Action Plan for the Prevention and Control of NCDs (2013–2030), *extended in 2020*	Governments were encouraged to “increase and prioritize budgetary allocations” for NCD prevention and control with a focus on best buy interventions (Appendix of the Global Action Plan).
**2013**	Establishment of the United Nations Interagency Task Force (UNIATF) on the Prevention and Control of Noncommunicable Diseases by the UN Secretary‑General	UNIATF brought together over 40 UN agencies for a whole‑of‑government and whole‑of‑society approach to scale up action to address NCDs. Since 2015, UNIATF has been working to achieve SDG target 3.4, UHC, and other health‑related SDGs.
**2014**	Establishment of the WHO Global Coordination Mechanism on the Prevention and Control of NCDs (GMC/NCD) by WHO (A67/14Add.1 8 May 2014)	The GCM/NCD Terms of Reference developed by the Member States confirmed one of the main functions of the GCM: the advocacy for resource mobilization. Other functions included advocacy to raise awareness, disseminate knowledge and information, identify barriers, encourage innovation, and advance multisectoral action.
**2014**	Second UNGA High‑Level Meeting on NCD Prevention and Control	“Continue to explore the provision of adequate, predictable, and sustained resources, through domestic, bilateral, regional, and multilateral channels, including traditional and voluntary innovative financing mechanisms.”
**2015**	Transforming Our World: The 2030 Agenda for Sustainable Development	Commitment to “substantially increase health financing.”
**2017**	WHO Global Conference on NCDs and Sustainable Development: Enhancing policy coherence between different spheres of policymaking that have a bearing on attaining SDG target 3.4 on NCDs by 2030 (Montevideo, Uruguay)	Montevideo Roadmap 2018–2030 committed to “increase significantly the financing of national NCD responses and international cooperation.”
**2018**	Report of the WHO Independent High‑Level Commission on NCDs: Time to Deliver	“Increase the percentage of national budgets allocated to health, health promotion, and essential public health functions, and within health, to NCDs and mental health.”
**2018**	First WHO Global Dialogue on Partnerships for Sustainable Financing of NCD Prevention and Control (Copenhagen, Denmark)	“Complement domestic resources, both public and private, with international cooperation, including official development assistance with a focus on LMIC, and other resources, to increase health expenditure on NCD prevention and control, consistent with country needs and priorities.”
**2018**	Third UNGA High‑Level Meeting on NCD Prevention and Control	Governments recommitted to “mobiliz[ing] adequate, predictable and sustained resources for national responses to prevent and control NCDs and to promote mental health and well‑being, through domestic, bilateral and multilateral channels, including international cooperation and development assistance.”
**2019**	First UNGA High‑Level Meeting on Universal Health Coverage (UHC): Universal Health Coverage—moving together to build a healthier world	Committed to “pursu[ing] efficient health financing policies…to eliminate barriers to access to quality, safe, effective, affordable and essential health services…throughout the life course, through better allocation and use of resources, with adequate financing for PHC, in accordance with national contexts and priorities.”
**2019**	WHO Global Meeting to Accelerate Progress on SDG Target 3.4 on NCDs and Mental Health (Muscat, Oman)	Focus on fiscal measures for health to ensure sustainable financing for NCD prevention and control and enhanced mental health and well‑being.
**2020**	Establishment of the WHO Global NCD Platform (GNP)	GNP was established to maximize synergies between GCM and UNIATF with a mandate to “coordinate the United Nations system and mobilize non‑State actors to unlock their transformative potential to complement and enhance WHO’s work in supporting governments to develop whole‑of‑government and whole‑of‑society responses to address SDG 3.4 and other NCD‑related SDGs.”
**2021**	Establishment of the UN Multi‑partner Trust Fund to address NCD and mental health conditions	The Multiparter Trust Fund (The Health4Life Trust Fund) led by UNIATF was established to catalyze countries’ action on NCDs and mental health.
**2021**	World Health Assembly Decision (WHA74(11)) to extend the WHO Global Coordination Mechanism on the Prevention and Control of NCDs until 2030, with five key areas of work	The role of the GCM/NCD in WHO’s work on multistakeholder engagement for the prevention and control of NCDs: (i) an operational backbone for knowledge collaboration and the dissemination of innovative multistakeholder responses at the country level; (ii) an enabler for the global stocktaking of multistakeholder action at the country level; (iii) the provision and updating of guidance to Member States on engagement with non‑State actors; (iv) a global facilitator for the strengthened capacity of Member States and civil society to develop national multistakeholder NCD responses; and (v) a convener of civil society, including people living with NCDs, to raise awareness and build capacity for their meaningful participation.
**2023**	Second UNGA High‑Level Meeting on UHCExpanding Our Ambition for Health and Well‑being in a Post‑COVID World	Recommitted to ensuring “a sustainable and adequate financing to implement high‑impact policies to protect and promote peoples health, including by providing financial risk protection, and addressing social, economic, environmental and other determinants of health by working across all sectors through health‑in‑all‑policies approach.”
**2024**	International Dialogue on Sustainable Financing to Address NCDs and Mental Health Conditions	Reiterated the need for “Increased and improved financing” to prevent and control NCDs and mental health conditions, including development assistance as a catalytic source of funding.

Table 1 provides a chronological overview of the global events and initiatives that consistently called for increased resource mobilization over the years to prevent and control NCDs and mental health conditions. The table was compiled through a review of high‑level political declarations, global action plans, and UN and WHO resolutions from 2011 onward, focusing on commitments explicitly referencing sustainable financing, resource mobilization, and investments in NCD prevention and control. All these initiatives called for increased resource mobilization to address NCDs and mental health conditions, underscoring governments’ leading role in ensuring sustainable healthcare, considering the demographic and epidemiological trends, and the contexts in which their healthcare systems operate. However, the implementation of these calls and commitments has proved to be challenging, particularly in low‑income settings.

## The Fourth UNGA HLM on NCD Prevention and Control in 2025: a Critical Opportunity

HLM 2025 is expected to produce a renewed political declaration to reaffirm the global commitment to reducing the burden of NCDs. By galvanizing global action and fostering a unified approach to tackling NCDs, the HLM 2025 is expected to build on previous work to significantly advance the global fight against these prevalent and preventable diseases, for improved health outcomes and sustainable development worldwide.

But what new commitments can be made in the political declaration? There is already a clear objective in the form of SDG target 3.4, an extensive updated list of highly cost‑effective policy options and interventions [10], and multiple UN declarations on the significance of NCDs, including high‑level commitments from all heads of state and government.

On the basis of the journey so far, we recommend that the following items feature in the political declaration:

First, the declaration should summarize the (inadequate) progress made against the 2025 and 2030 global targets, providing quantitative data broken down by gender and region. Second, the declaration should set out clear and measurable targets for the next five years, plotting an achievable course to the attainment of SDG 3.4 for each region. Different countries will require different approaches, and the meeting should focus on identifying credible pathways to achieving global targets for countries facing similar challenges. Third, the declaration should include a greater focus on increasing investments in NCD prevention and control by dedicating a higher amount of funds to health and introducing cross‑sectoral financing, where money will be pooled across relevant public sectors and then used to purchase healthcare and non‑health interventions to address NCDs and their underlying determinants [11].

However, what is the right amount of spending that countries need to commit to, and what are the priority areas for investment that will yield the greatest benefits?

Various reports have proposed a range of targets for governments’ spending on health. The best known is the African Union’s commitment, reflected in the Abuja Declaration, to devote 15% of government budgets to health. Others have argued that “a target of government expenditure of at least 5% of GDP is an appropriate one” [12]. Allocation of funds to NCD prevention and control is particularly challenging in LMICs with many competing priorities. It is estimated that low‑income countries (LICs) allocate about 13% of public health expenditure to NCDs, while middle‑income countries (MICs) dedicate approximately 30% of overall spending to health [13]. In LMICs, addressing NCDs will require approximately USD 140 billion in new spending over 2023–2030, averaging USD 18 billion annually. These investments have the potential to avert up to 39 million deaths over this period and generate USD 2.7 trillion in net economic benefits [14].

Achieving SDG target 3.4 will require targeted action on NCDs and mental health conditions in all countries. The WHO best buys and other recommended interventions provide an excellent menu of policy options encompassing clinical and public health strategies to address NCDs and their risk factors [10]. However, their implementation will require increased investments to address the following challenges related to governance, health service delivery, multisectoral action, vulnerable populations, and implementation research.

1. Strengthening Ministries of Health
  Ministries of Health have a critical role in ensuring policy coherence with other sectors and facilitating effective service delivery in any country, regardless of the country’s level of development. However, to ensure the coordinated action of a wide range of stakeholders, Ministries of Health need human and financial capacities to take up the challenges. The competencies necessary to work beyond the health sector and engage non‑state actors, particularly the private sector, are of particular significance owing to potential conflicts of interest that may not benefit public health, especially the NCD agenda.
2. Integrated Care
  NCDs and mental health conditions are lifelong chronic conditions requiring regular and timely access to care. An integrated approach to delivering a continuum of health promotion, disease prevention, diagnosis, treatment, disease control, rehabilitation, and palliative services across the levels of care, delivery platforms, and life course to address NCD risk factors and the underlying determinants has the potential to reduce premature mortality and morbidity from chronic diseases, strengthen health systems for universal health coverage, and increase the efficiency and effectiveness of services [15]. Integration of NCDs into other program areas, such as human immunodeficiency virus (HIV)/acquired immunodeficiency syndrome (AIDS), tuberculosis (TB), and maternal and child health programs can also maximize synergies and improve access to and quality of care.
  An integrated approach can reduce the risk of fragmentation in care and ensure that high‑risk populations receive continuous lifelong support. Recent findings from the WHO confirm that integrated health services lessen the burden of premature mortality from NCDs and mental health conditions, especially in under‑resourced settings [16]. However, to provide such a broad range of integrated services, substantial investments will be needed in an interdisciplinary health workforce, particularly at the primary health care level (PHC) to equip them with the necessary skills and competencies to address a wide spectrum of services, including the prevention and management of NCDs, their risk factors, and mental health conditions [17].
3. Vulnerable Populations
  While population‑level interventions seek to improve overall health, they may still fail to support vulnerable populations [18]. NCDs affect different groups, such as people living below the poverty line; older persons; people living with HIV, TB, and other multimorbidities and disabilities; refugees; the internally displaced; migrants; and other marginalized populations, differently. These populations usually have insufficient resources to cope with health challenges. Prioritizing health investments targeting vulnerable groups leads to improved economic stability, increased workforce productivity, and substantial reductions by mitigating the burden of chronic diseases [19]. Investing in these groups contributes to better health outcomes but also serves to create more resilient and equitable societies that are better placed to resist economic and social shocks, highlighted once again by the coronavirus disease 2019 (COVID‑19) pandemic. The focus on these populations also aligns with SDG 10 on reducing inequalities. Sustainable financing of health systems that meet the needs of vulnerable populations is essential for achieving SDG 3.4, which targets the reduction of premature mortality from NCDs. Robust health information systems are critical to conduct disaggregated data collection and analysis of NCD prevalence and risk factors among vulnerable groups.
  Finally, the involvement of people living with NCDs, mental health conditions, and neurological conditions in all aspects of the NCD and mental health responses that affect them, including the development of policies, programs, and services, is paramount. Without their engagement, active participation, and contribution, sustainable progress cannot be made.
4. Multisectoral Action and Collaboration
  Investment in multisectoral action for UHC is no longer an option but a necessity to respond to the complex and often interrelated health problems that countries face today. The need to scale up multisectoral cooperation is also urgent owing to converging global health agendas [11]. A whole‑of‑government approach can facilitate the alignment of all health goals and make resources and actions across sectors more efficient in dealing with common root causes of health problems. This investment, therefore, is valuable because every dollar works harder by avoiding duplication and addressing overlapping social determinants of health [11–16].
  We distinguish between multisectoral and multistakeholder collaboration, the former referring to policy coherence and coordination across different public sectors, and the latter to the engagement with non‑state actors, such as civil society and the private sector. Collaborating with the private sector to tackle NCD risk factors can drive policy change, enhance resources, and foster innovation [20]. However, it is essential to establish safeguards for engagement rules, align incentives with public health goals, and manage conflicts of interest [20]. WHO offers resources on effectively engaging with the private sector for the prevention and control of NCDs to avoid any potential conflict of interest jeopardizing the achievement of public health goals [21]. We have previously elaborated on the practical mechanisms of funding multisectoral actions such as intersectoral co‑financing, targeted investment incentives within UHC frameworks, and international donors [11]. These collaborative funding approaches to investing in health will tackle the root causes of ill health and, eventually, lead to fairer and stronger health systems for everyone.
5. Implementation Research
  More investments will be needed in implementation research, which encompasses the tools of both implementation science and health policy and systems research to identify best practices and context‑specific solutions to prevent and control NCDs and mental health conditions [22]. The WHO’s mid‑point evaluation of the Global Action Plan (GAP) on NCDs (2013–2030) [23] highlighted a critical funding gap in this area, which limits countries’ capacity to adapt and translate global policies into country‑level policies and programs.
  In 2020, global grant funding for health research totaled USD 37 billion, with LICs receiving just 0.2% (USD 85 million) and MICs and upper‑middle‑income countries (UMICs) each receiving 0.5% (USD 188 million and USD 193 million, respectively). High‑income countries (HICs) received most of the funding, indicating a significant imbalance [24]. NCDs in LICs and LMICs received just 0.2% (USD 48 million) of the USD 21.4 billion allocated for NCD research, despite their significant burden, and the total funding has remained nearly the same since 2016 for these countries [24]. The investment in health research also differs greatly among the WHO regions [25].
  In 2021, the WHO reported that under 50% of countries in each region had NCD‑related research policies or plans, but only one in five countries had a national network for NCD‑related research and evaluation, with two‑thirds of these networks found in the Eastern Mediterranean and European regions [26].
  Greater investments in implementation research will help countries develop solutions that are feasible to implement and suit their national contexts [23].

## Conclusions

The investments needed for the prevention and control of NCDs and enhanced mental health are a health imperative, but also a key political decision that will determine the future sustainability of economies and the resilience of societies. Despite the abundant evidence on the cost‑effectiveness of interventions to manage NCDs, these conditions remain underfunded and often deprioritized in comparison with other global health areas.

At best, the upcoming 2025 UN High‑Level Meeting on NCDs provides an opportunity to galvanize action and establish clear strategies and targets to meet global commitments on NCDs, tailored to the needs of individual countries. At worst, the same platitudes will be rehearsed without any real commitment or substance. Specific, measurable, actionable, and timebound commitments are required, along with enhanced accountability mechanisms and increased levels of financial and technical support. Sustainable health financing, particularly in LMICs, remains key—not just increased domestic spending, but innovative international partnerships and pooled resources, with an emphasis on preventive and integrated care models. Reaffirming and expanding commitment to health equity targeting investments toward the most vulnerable populations and strengthening health systems must be done if any real progress is to be made toward attaining SDG target 3.4.
